# How I prescribe continuous renal replacement therapy

**DOI:** 10.1186/s13054-020-03448-7

**Published:** 2021-01-02

**Authors:** Emily J. See, Rinaldo Bellomo

**Affiliations:** 1 Department of Intensive Care, Austin Hospital, 145 Studley Road, Heidelberg , VIC Australia; 2 Department of Nephrology, The Royal Melbourne Hospital, Parkville, Australia; 3 Centre for Integrated Critical Care, University of Melbourne, Melbourne, Australia; 4 Department of Intensive Care, The Royal Melbourne Hospital, Parkville, Australia; 5 Data Analytics Research and Evaluation, The University of Melbourne and Austin Hospital, Melbourne, Australia

## Introduction

**Fig. 1:**
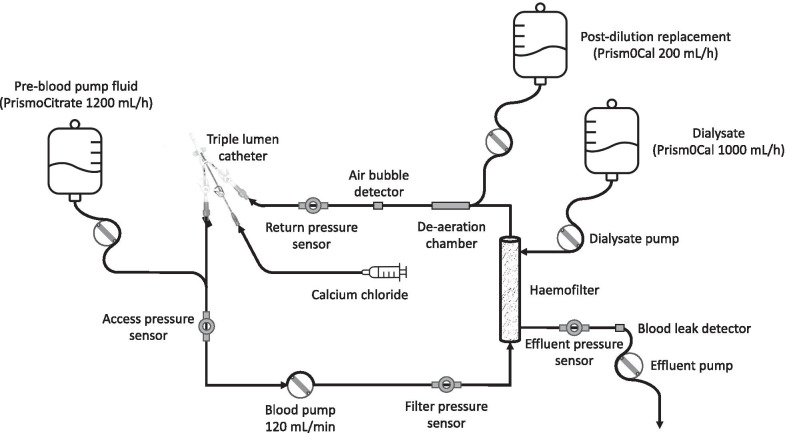
Our typical CVVHDF circuit with regional citrate anticoagulation. This circuit can be altered for CVVH by removing the administration of dialysate or for CVVHD by removing the administration of pre-blood pump fluid and post-dilution replacement fluid

Continuous renal replacement therapy (CRRT) delivers gradual clearance of solutes, fluid balance control, and haemodynamic stability. CRRT does not appear to increase survival compared to intermittent renal replacement therapy (IRRT), but may affect renal recovery [1, 2]. Here, we describe how we prescribe CRRT (Fig. 1).

## Timing of initiation

Early CRRT initiation may not improve outcomes, and the definition of “early” varies between studies [3–5]. Therefore, clinical judgement guides CRRT initiation. We aim to prevent or rapidly treat life-threatening derangements in fluid status, electrolytes, and/or acid–base balance and to meet metabolic and fluid needs that residual kidney function cannot address.

## Catheter selection

Catheters should be of sufficient gauge (13 Fr or 13.5 Fr) to deliver the desired blood flow rate without high negative pressures. The insertion site depends on clinical judgement. Catheter function is best with the right internal jugular vein, followed by femoral vein, and left internal jugular vein [6]. We avoid any other lines in the same vessel and the subclavian vein due to the risk of thrombosis or stenosis. Targeting soft tip position in the right atrium or in the proximal inferior vena cava helps maximise circuit life [7]. Triple lumen catheters (13 Fr) facilitate calcium administration during citrate anticoagulation. However, blood for ionised calcium measurements should come from the arterial line. Avoiding femoral access in obese patients may decrease catheter-related bloodstream infections [8].

## Anticoagulation

The risks of clotting and bleeding must be carefully considered. Regional (e.g. citrate-calcium or heparin-protamine) or systemic anticoagulation approaches (e.g. unfractionated heparin, low molecular weight heparin) are available. Regional citrate anticoagulation reduces the risk of circuit loss, filter failure, bleeding, and heparin-induced thrombocytopaenia [9]. Accordingly, we prescribe regional citrate anticoagulation. We avoid citrate in patients with severe liver failure or a serum lactate > 4 mmol/L due to the risk of citrate intolerance. In acute liver failure, we typically perform CRRT without anticoagulation.

## CRRT modality

There are three key equivalent CRRT modalities (Fig. 1): Continuous venovenous haemofiltration (CVVH); continuous venovenous haemodialysis (CVVHD); and continuous venovenous haemodiafiltration (CVVHDF) [10]. Accordingly, modality selection is based on local expertise. We preferentially prescribe CVVHDF because it is the most well studied and because diffusion may prolong circuit life [11, 12].

## CRRT dose

CRRT dose is essentially quantified by the effluent flow rate and there is no survival benefit from a dose > 20 to 25 mL/kg/h [12]. We prescribe an effluent flow rate of 25 mL/kg/h to achieve a delivered dose of at least 20 mL/kg/h. Patients with severe metabolic derangements may benefit from higher CRRT dosage [13]. In patients with hyperammonaemia (> 100 µmol/L), we prescribe 50 mL/kg/h of effluent flow rate to target levels < 100 µmol/L. We prescribe a similar intensity CRRT for severe hyperkalaemia.

## Blood flow rate

Blood flow rate prescription varies with modality. For CVVHD, the blood flow rate should be at least twice the dialysate flow rate to maximise the plasma to dialysate concentration gradient. For CVVH, blood flow rate should be titrated to prevent a filtration fraction (plasma water removal to plasma flow ratio) > 25%. Pre-filter replacement fluid administration requires adjustment to this calculation. We reach our target blood flow rate in a stepwise manner starting at 25 mL/min and increasing slowly (over 10–15 min). Once established, typical blood flow rates (150–250 mL/min) do not affect haemodynamics. For citrate CVVHDF, we prescribe a lower blood flow rate of 120 mL/min because higher rates necessitate a higher dose of citrate, which increases the risk of citrate toxicity. We do not change blood flow rate according to pre vs. post filter replacement fluid administration, despite differences in solute clearance efficiency.

## CRRT solutions

Bicarbonate-buffered solutions are preferred over lactate-buffered solutions to prevent iatrogenic hyperlactataemia. Phosphate-containing solutions are available. Although effective at preventing hypophosphataemia, they increase the risk of hypocalcaemia and metabolic acidosis [14]. For patients receiving regional citrate anticoagulation, we use commercially available pre-blood pump fluid containing citrate as well as calcium-free dialysate and post-dilution replacement fluid to preserve the anticoagulant effect of citrate. We administer the majority of replacement fluid pre-filter to deliver the prescribed dose of citrate into the circuit. With citrate, we use lower bicarbonate replacement fluids (22 mEq/L) with either 0 or 4 mmol/L of potassium, depending on serum potassium levels.

## Patient fluid removal

The difference between ultrafiltration and replacement/dialysate volumes determines fluid removal. The speed of fluid removal is referred to as the net ultrafiltration (NUF) rate. A high NUF rate in CRRT may be harmful [12], although optimal values are not yet established. Because fluid overload is common and undesirable, we regularly reassess fluid status and adjust NUF rate accordingly. We avoid very high NUF rates (> 2 mL/kg/h), unless aggressive fluid removal is indicated by life-threatening fluid overload.

## Monitoring therapy

We monitor electrolytes every 6–8 h. We measure ionised calcium, total calcium and plasma bicarbonate 4–6 hourly in patients receiving regional citrate anticoagulation. In keeping with Acute Dialysis Quality Initiative (ADQI) recommendations, we audit CRRT safety and quality by monitoring circuit life, small-solute clearance, delivered dose, catheter dysfunction, catheter infection, and mortality [15].

## When to stop

The decision to discontinue CRRT is based on clinical judgement. However, higher urine output, higher creatinine clearance, and lower serum creatinine can predict successful CRRT cessation [5]. A trial of CRRT cessation is appropriate when spontaneous urine output is > 500 mL/day and endogenous creatinine clearance is > 15 mL/min. We delay exposure to IRRT until at least 24 h after cessation of vasopressor drugs.

## Conclusions

We prefer CVVHDF with regional citrate anticoagulation via a triple lumen catheter inserted into the right internal jugular vein or the right femoral vein. Timing of initiation and cessation of CRRT is based on clinical judgement. We prescribe a blood flow rate of 120 mL/min and an effluent flow rate of 25 mL/kg/h with citrate anticoagulation. We avoid aggressive NUF unless clinically indicated. We adjust effluent flow rate for specific patients to target ammonia clearance. We monitor the safety and quality of CRRT and advocate for the use of protocolised care. Although intensivists prescribe CRRT in our unit, we acknowledge that collaborative and multidisciplinary prescription is common worldwide.

## Data Availability

Not applicable.
